# Correlation between the Compressive Strength and Ultrasonic Pulse Velocity of Cement Mortars Blended with Silica Fume: An Analysis of Microstructure and Hydration Kinetics

**DOI:** 10.3390/ma14102476

**Published:** 2021-05-11

**Authors:** Geuntae Hong, Sangwoo Oh, Seongcheol Choi, Won-Jong Chin, Young-Jin Kim, Chiwon Song

**Affiliations:** 1 Department of Civil and Environmental Engineering, Chung–Ang University, 84 Heukseok-Ro, Dongjak-Gu, Seoul 06974, Korea; hgt0916@naver.com (G.H.); dhfflqj8@naver.com (S.O.); schoi@cau.ac.kr (S.C.); 2 Structural Engineering Research Institute, Korea Institute of Civil Engineering and Building Technology, 283 Goyangdae-Ro, Ilsanseo-Gu, Goyang-Si 10223, Gyeonggi-Do, Korea; wjchin@kict.re.kr (W.-J.C.); yjkim@kict.re.kr (Y.-J.K.)

**Keywords:** compressive strength, hydration kinetics, microstructure, silica fume, ultrasonic pulse velocity

## Abstract

The effect of the replacement rate of silica fume (SF) on the correlation between the compressive strength and ultrasonic pulse velocity (UPV) of cement mortar was experimentally analyzed. Specimens were fabricated with different replacement rates of SF, the compressive strength and UPV were measured, and isothermal calorimetry and mercury intrusion porosimetry tests were conducted to analyze the effects of replacement on the hydration kinetics and microstructures on these properties. Field emission scanning electron microscopy analysis was performed to observe SF particles and microstructure. The substitution of SF changed the cement mortar’s hydration kinetics and microstructures, resulting in different strengths and UPVs depending on the replacement rate. The compressive strength and UPV for cement mortars blended with SF also showed a different exponential relationship depending on the SF replacement rate.

## 1. Introduction

With the need for CO_2_ reduction catching up with the cement industry, various studies [1,2,3,4] on its industrial by-products (e.g., fly ash, ground granulated blast-furnace slag, and silica fume) have been conducted. These industrial by-products, supplementary cementitious materials (SCMs), have been reported to improve the service life of concrete structures because they significantly improve strength and durability by changing the microstructures of the cement matrix. In particular, silica fume (SF) is one of the most effective SCMs in improving the durability and mechanical performance of cementitious materials. Recently, the increasing demand for long-span bridges and high-rise buildings has resulted in an increase in the research in this area [5,6,7,8,9,10,11,12]. Perraton et al. experimentally demonstrated that the chloride ion diffusion in concrete significantly decreased as the amount of SF added increased [7]. Dotto et al. suggested that the addition of SF in concrete has a great effect in protecting against corrosion of the reinforcement bars [8]. Although results have differed among researchers, it is generally reported that the substitution of an appropriate amount of SF increases the compressive strength and tensile strength of cementitious materials at all ages [9,10,11,12]. The improvement in durability and mechanical performance of cementitious materials by SF is caused by the following factors: pore-size refinement, cement-matrix densification, reaction with free lime, and interfacial transition-zone refinement [13,14,15].

The mechanical properties of cementitious materials blended with SF are sensitive to environmental factors, mixture proportions, raw-material properties, and age, compared with those of normal cementitious materials [16,17]. Therefore, using SF-blended cementitious materials at an actual construction site requires a technology that can accurately estimate the mechanical properties. Compressive strength that changes with age can be estimated by using the ultrasonic pulse velocity (UPV) test. The UPV test is a non-destructive test that can estimate the compressive strength and detect defects in concrete and can be easily applied in both laboratory and field environments. Thus, several studies [18,19,20,21,22] on its efficacy have been conducted, and estimations of compressive strength, elastic modulus, and setting have been carried out using the UPV method [20,21,22,23,24]. Demirboğa et al. assessed the relationship between compressive strength and UPV according to the substitution amount of blast-furnace glass powder and fly ash [22]. ABO–Qudais analyzed the relationship between the compressive strength and UPV of concrete with different water-to-cement ratios and aggregate gradations [23]. Ashrafian et al. evaluated the compressive strength of fiber-reinforced concrete incorporating nano-silica via UPV [24]. Additionally, studies have examined the relationship between compressive strength and UPV for cementitious materials in terms of mixture proportions. UPV is closely related to the mechanical properties and microstructures of cementitious materials [25,26,27], both of which are greatly influenced by the hydration reaction between the binder materials and water. Therefore, the effect of the replacement rate of SF on the correlation between compressive strength and UPV should be analyzed from a material point of view.

This study aimed to comprehensively investigate various factors related to the compressive strength and UPV of cement mortars blended with SF and to analyze their correlation. To this end, cement mortar specimens with SF replacing other binder materials at replacement rates of 0, 10, 20, and 30% were fabricated. The compressive strength and UPV of each specimen were measured at 1, 3, 7, 14, and 28 days of age. The microstructure and hydration kinetics were analyzed by mercury intrusion porosimetry (MIP) and isothermal calorimetry experiments, respectively. The variation in the compressive strength and UPV of each specimen and their correlation were determined. Field emission scanning electron microscopy (FE-SEM) analysis was also performed to evaluate the effect of age and SF addition on the microstructural change of the cement matrix.

## 2. Experiment

### 2.1. Materials and Mixture Proportions

**Figure 1:**
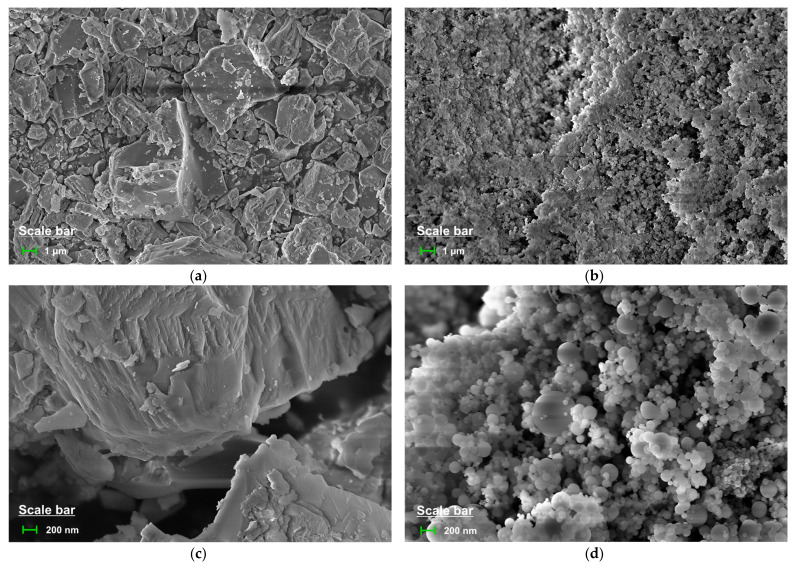
SEM images of binders: (**a**) Cement (magnification of 10,000×); (**b**) Silica fume (magnification of 10,000×); (**c**) Cement (magnification of 50,000×); (**d**) Silica fume (magnification of 50,000×).

**Figure 2:**
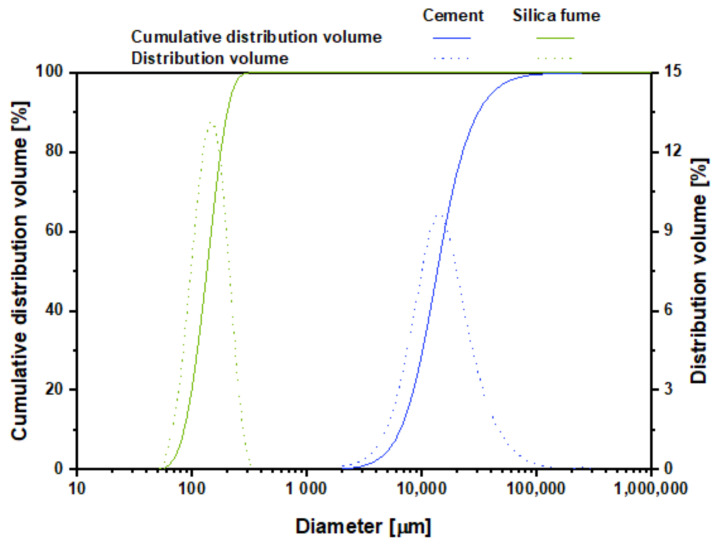
Particle-size distributions of the binder materials.

Type-I Portland cement (Ssangyong Cement Industrial Co. Ltd., Seoul, Korea) and SF (Elkem Materials Co. Ltd., Kristiansand, Norway) were used as binder materials. The SiO_2_ content of SF was 93.1%. The surface areas of the cement and SF were 3300 and 217,000 cm^2^/g, respectively. Table 1 shows the chemical compositions of the binder materials. Figure 1 and Figure 2 show the SEM images and cumulative particle-size distribution of the binder materials. It can be seen that the particle size of SF is very small compared to that of cement. The fine aggregate was standard sand with more than 98% of SiO_2_ by mass. A polycarboxylic acid chemical admixture (Rheobuild SP8HU (SR), BASF Japan Ltd., Tokyo, Japan) was used to enhance the workability.

Table 2 shows the cement mortar mixture proportions with a binder weight of 1000 g. The variations in the mixture proportions are in replacement rates of SF at 0, 10, 20, and 30% (by binder replacement). The ratios of binder-to-sand and water-to-binder are 1.0 and 0.25, respectively. To minimize the effect of the chemical admixture, the amount added was equal to 2.0% of the binder weight in all specimens. In Table 2, the numbers following the letter “S” in the ID of the specimen indicate the replacement rate of SF by the weight of the binder.

### 2.2. Test Methods

Dry cement, SF, and sand were mixed for 5 min to ensure a homogenous distribution. Next, water and chemical admixture were added to the mixture and mixed at a low speed (140 ± 5 rev/min) for 3 min. The wall of the mixer bowl was scraped to prevent heterogeneous mixing by the mixture attached to the wall, and the fresh cement mortar mixtures were mixed at an intermediate speed (285 ± 10 rev/min) for 5 min. The mixtures were then cast into molds.

The compressive strengths of the cement mortar specimens were measured at different ages (1, 3, 7, 14, and 28 days) in accordance with the ISO 679 standard. After casting the fresh cement mortar mixtures into a 40 × 40 × 160 mm^3^ beam-shaped mold, they were cured at room temperature (23 ± 1 °C) under sealed conditions, using aluminum foil tape, to prevent moisture escaping. Upon completion of curing, the specimens were split in half in the transverse direction and the compressive strength was measured. The arrival time of the ultrasonic pulse was measured with a cylindrical specimen having a size of 100 × 200 mm^2^ using the Ultracon-170 ultrasonic detector (MKC Korea, Seoul, Korea) for UPV analysis. The transducer has a frequency of 52 kHz. The specimen was cured in a sealed state in the same environment as the compressive strength measurement specimen.

To measure the heat evolution rate of the specimens with age, an isothermal calorimetry test was conducted using a TAM-AIR isothermal calorimeter (TA Instruments, New Castle, DE, USA) in accordance with ASTM C1702 standards. After mixing fresh cement mortar, approximately 4 g of the mixture was put into a glass ampoule, and the isothermal calorimetry test was conducted at room temperature for 28 days. The MIP test for microstructure analysis was performed using the specimens fractured after measuring the compressive strength. The specimens were cut into 10 × 10 × 10 mm^3^ cuboid shapes. Subsequently, the specimens were immersed in an acetone solution for 24 h at room temperature in order to stop the hydration reaction. Thereafter, the specimens were placed in an oven at 60 °C for 24 h to remove acetone and moisture from the specimens. Finally, measurements of total porosity and pore-size distributions of the specimens at 1, 3, 7, 14, and 28 days of ages were performed using Micromeritics’s AutoPore IV9500 (Micromeritics Instrument Corp., Norcross, GA, USA). In addition to the MIP test, FE-SEM (Carl Zeiss, Sigma, Oberkochen, Germany) analysis was also performed on a fractured specimen to confirm the microstructure of the cement matrix after measuring the compressive strength in the same manner as the MIP test. The FE-SEM used in this study has the advantage of less radiation damage to the sample because it can observe high magnification at a low-speed voltage. The specimens were cut into 1 × 1 mm^2^ thin film forms, which were immersed in acetone solution and dried in an oven. Subsequently, their surfaces were coated with Pt prior to the SEM analysis.

## 3. Results and Discussion

### 3.1. Hydration Kinetics and Microstructures

**Figure 3:**
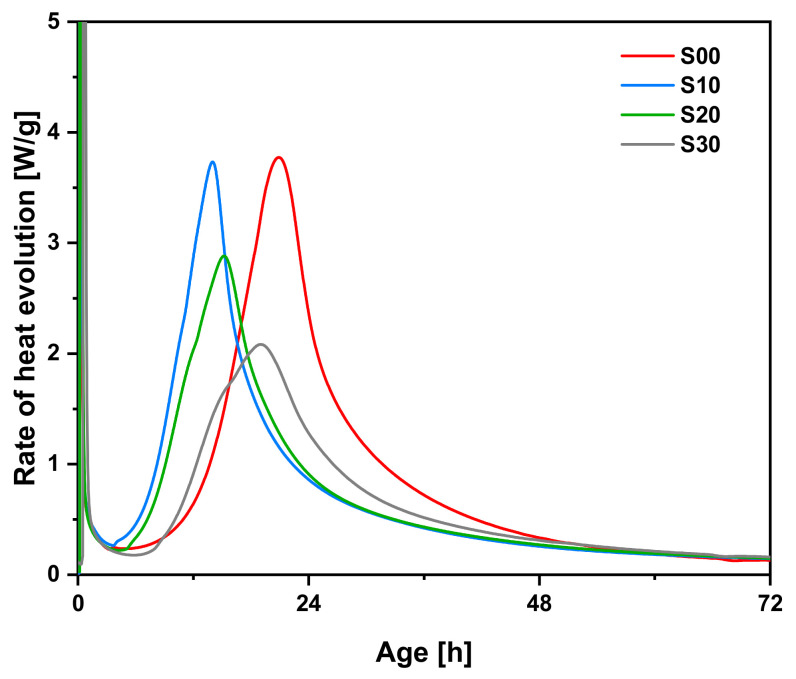
Rate of heat evolution of cement mortars blended with SF up to 72 h of age.

Figure 3 shows heat evolution rate curves for each specimen until 72 h. The curves generally consist of five stages: initial reaction, induction period, acceleration period, deceleration period, and a period of slow continuous reaction [28]. The peaks in the initial reaction appeared within minutes after the cement and water were mixed. In the induction period, the heat evolution rate rapidly decreases; however, heat is still generated by the hydration reaction, which is related to calcium silicate hydrate (C–S–H) formation with a low Ca/Si ratio as a protective layer over the surface of the cement clinker particles. The layer is destroyed at the end of this period, and various ions begin to be eluted from the surface of the unhydrated cement and enter an acceleration period, during which the silicate eluted from the cement particles reacts rapidly and the rate of heat evolution reaches its peak following the initial reaction. The rate of heat evolution occurring during the acceleration period is influenced by the formation rate of C–S–H [29]. The magnitude of the peak in the acceleration period tended to decrease as the SF replacement rate grew. The peak values for S00, S10, S20, and S30 were 3.77 mW/g at 20.87 h, 3.73 mW/g at 14.01 h, 2.88 mW/g at 15.19 h, and 2.08 mW/g at 19.02 h, respectively. The appropriate replacement of SF accelerates heat evolution by activating nucleation in the pore solution [30,31]. In addition, as SF has pozzolanic reaction characteristics, it does not react chemically at an early age. Therefore, it appears that the replacement of SF reduces the rate of heat evolution of cementitious materials at an early age [32]. The cement mortar specimens blended with SF are more dominantly affected by the latter, making the peak value reduce gradually during the acceleration period as the SF replacement rate increased. Increased SF replacement also shortened the induction period. During the first few minutes of the hydration reaction, alkali ions and Ca^2+^ ions are rapidly released from the cement particles. SF particles adsorb calcium ions on the surface and reduce the concentration of Ca^2+^ in the pore solution, boosting the dissolution of C_3_S and accelerating the hydration reaction [33]. After the induction ends, the cement particles are covered with hydration products, and the hydration reaction gradually slows. The rate of heat evolution is affected mainly by ion diffusion during this period.

**Figure 4:**
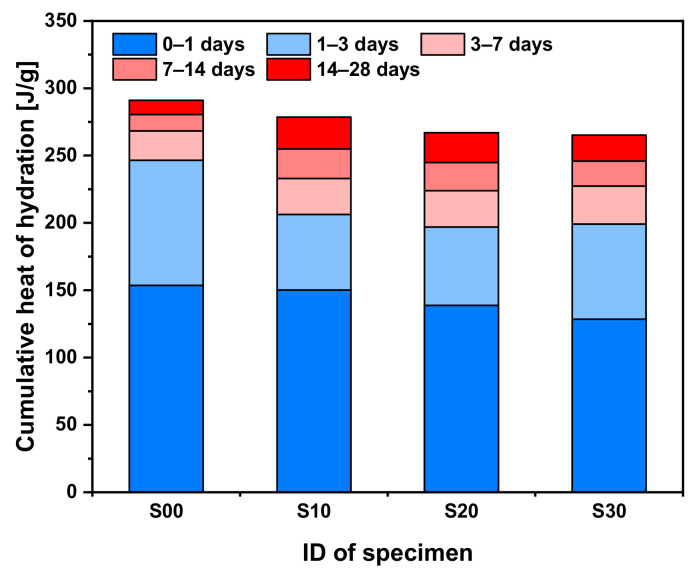
Cumulative heat of hydration of cement mortars blended with SF.

Figure 4 shows the cumulative heat of the hydration reaction. Across the specimens, the heat of hydration was produced mostly before 3 days of age, and the amount of heat generated gradually decreased with age. As mentioned earlier, SF in the early ages merely acts as a filler in the cement matrix and does not react chemically; therefore, the heat evolution during the acceleration period gradually decreased as the SF replacement rate increased. Therefore, as the amount of replacement increased, the cumulative heat of the hydration value tended to decrease at early ages. However, due to the pozzolanic reaction of SF, the hydration heat soared gradually with increasing SF replacement rates after 3 days of age. The rates between the cumulative heat of hydration at 3 days and that at 28 days were 84.72% in S00, 74.06% in S10, 73.80% in S20, and 75.08% in S30. At 28 days of age, the cumulative heat of hydration for the S00 specimen was the highest at 290.99 J/g, and the values for S10, S20, and S30 specimens were 95.72, 91.74, and 91.14%, compared with the S00 specimen. 

**Figure 5:**
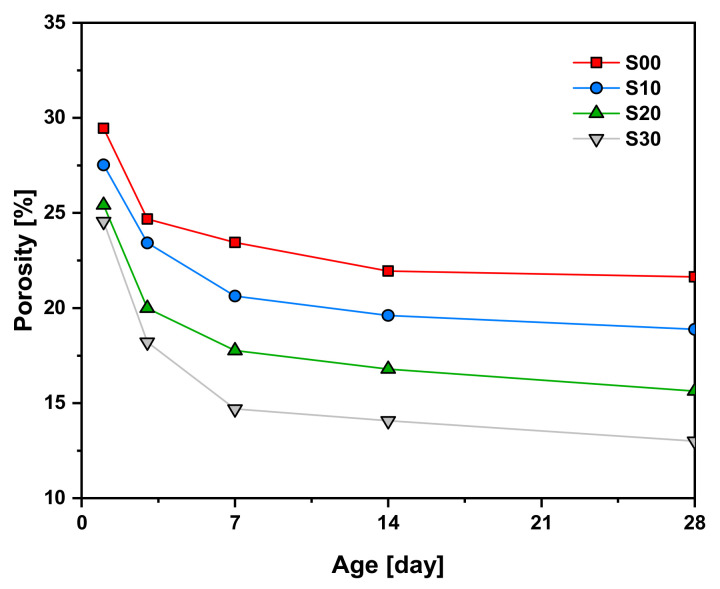
Total porosities of cement mortars blended with SF.

Figure 5 shows the variation in total porosity of each specimen at 1, 3, 7, 14, and 28 days of age. As the age increased, the hydration and the pozzolanic reaction of the cement and the SF gradually reduced the total porosity by filling the pores with hydration products. As the replacement rate of SF increased, the porosity decreased, and this tendency became more pronounced at later ages. At 1 d of age, the porosities of S10, S20, and S30 specimens were −1.93, −4.04, and −4.91, respectively, compared with that of S00 specimens. However, at 28 days of age, the difference was −2.76, −6.01, and −8.65, respectively. In other words, the porosity of cement mortar decreased at an early age as the SF replacement rate increased, and this trend became larger at a later age. At an early age, SF reduces porosity by acting as a simple filler in the cement matrix. However, at a later age, the SF acts as a filler and reacts with CH hydration product produced by the hydration of cement to form additional C–S–H. As seen above, this leads to an increase in the total cumulative heat of hydration, reducing porosity more intensely at later ages.

**Figure 6:**
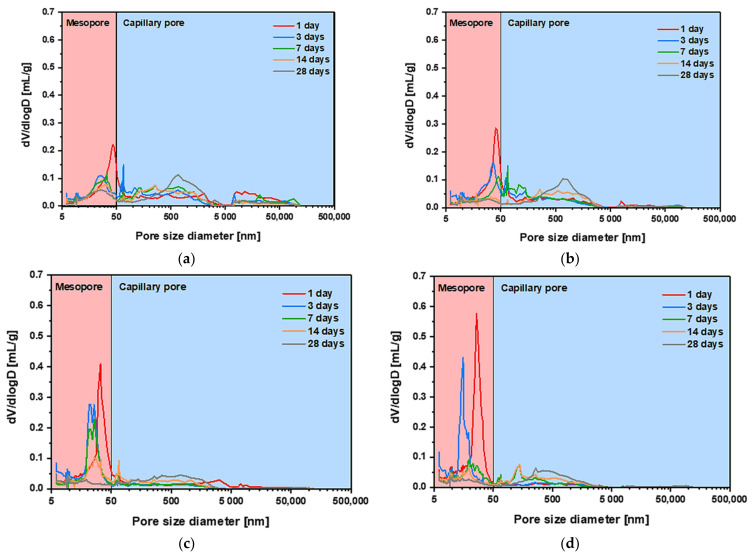
Pore-size distribution of cement mortar blended with SF: (**a**) S00 specimen; (**b**) S10 specimen; (**c**) S20 specimen; (**d**) S30 specimen.

Figure 6 shows the variation in the differential pore-size distribution curve with age. The pores in cementitious materials with porous properties are divided according to their diameter and, in this study, they were divided into mesopores (6–50 nm) and capillary pores (>50). In all specimens, peaks occurred in the mesopore range with diameters between 25–50 nm at 1, 3, and 7 days of age. The magnitude of the peak gradually decreased as products from SF’s pozzolanic reaction and the hydration reaction of cement-filled the pores in the cement matrix with age. In addition, the magnitude of the diameter peak tended to increase rapidly with increasing SF replacement rates. This is caused by the refinement of the microstructure, owing to the filling effect of the SF in the cement matrix [15,34]. The peak diameter value gradually decreased with age. The differential pore-size distribution curve was flattened and did not have a peak as the replacement rate of SF increased. This phenomenon is caused by the pozzolanic reaction of SF. The bolstered hydration effect due to the pozzolanic reaction of the SF becomes more pronounced as the age increases. Therefore, as the replacement rate of SF and age increase, the pores in the empty state are filled with hydration products, resulting in a flat differential pore-size distribution curve. Additionally, the peak values in the mesopores increased with the SF replacement rate at early ages, but the peak in the capillary pores, which significantly affects the mechanical properties of cementitious materials, decreased. Later in age, as the replacement rate of SF increased, the magnitude of the observed peak values tended to decrease overall, regardless of pore size, owing to further hydration effects of the SF.

### 3.2. Compressive Strength

**Figure 7:**
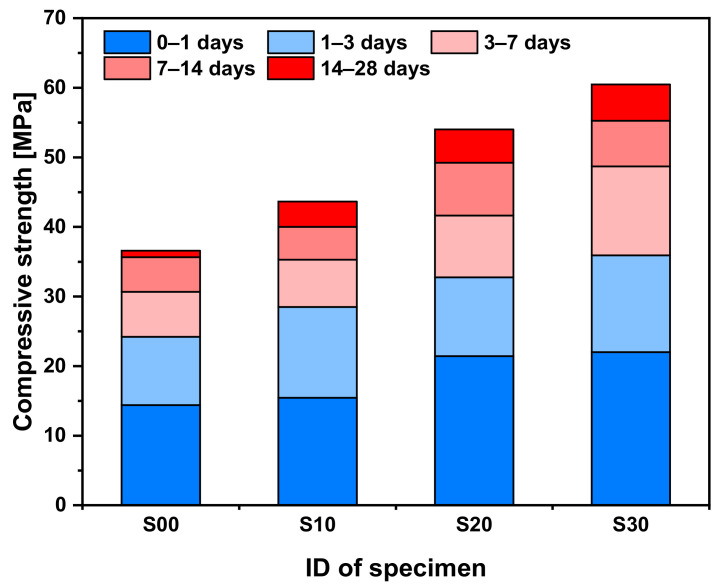
Compressive strength of cement mortar blended with SF.

Figure 7 shows the development of compressive strength with age for each specimen. The compressive strength gradually increased due to the change in the microstructure with age. Additionally, the compressive strength tended to increase at all ages with the replacement rate of SF, and, in particular, the compressive strength increase rate was largest at later ages. Compared to the compressive strength of the S00 specimen, the 3 days strength of the S10, S20, and S30 specimens increased by 9.86, 26.25, and 38.47%, respectively, and the 28 days strength increased by 11.99, 38.54, and 55.14%, respectively. This result is observed because the microstructure of the cement matrix becomes denser, owing to the pozzolanic reaction of SF (refer to Figure 4 and Figure 5). SF acts as a simple filler in the cement matrix at an early age, thereby increasing the compressive strength of the specimen by dispersing the load concentration. As the hydration products filled the pores via further hydration from the pozzolanic reaction as well as the role of SF as a filler, densification was amplified, resulting in a higher strength enhancement effect [35].

**Figure 8:**
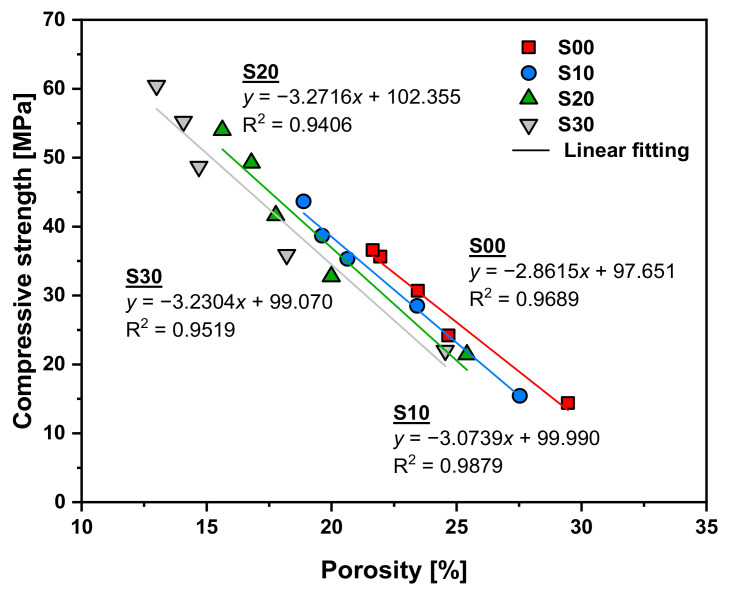
Compressive strength as a function of porosity.

**Figure 9:**
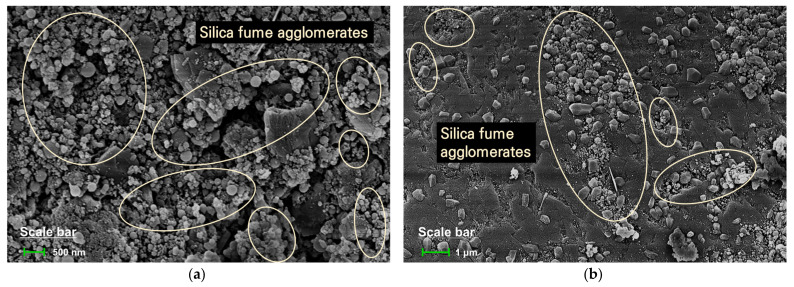

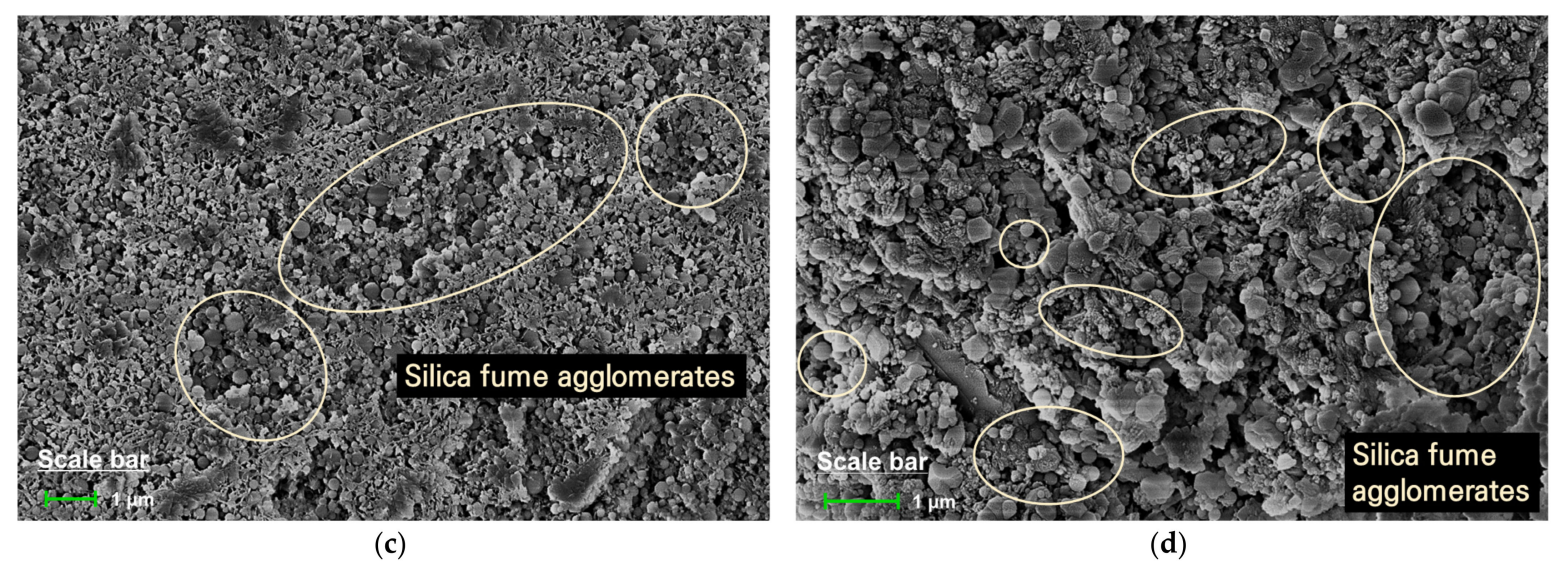
SEM images of SF particles in the cement matrix: (**a**) S10 specimen at 3 days; (**b**) S10 specimen at 28 days; (**c**) S30 specimen at 3 days; (**d**) S30 specimen at 28 days.

Figure 8 shows the results of a regression analysis on the correlation between compressive strength and porosity. The compressive strength of the specimens varies inversely with porosity, which is consistent with the results of other studies [36,37]. Compressive strength and porosity show a direct linear relationship in all specimens. The correlation coefficient for the relationship between compressive strength and porosity is higher than 0.9406. The linear slope reflects the increase in compressive strength caused by the decrease in porosity, and there are slight differences for each specimen. As the replacement rate of SF increases, the absolute value of the linear slopes tends to gradually increase. This indicates that even with the same level of decrease in porosity, the compressive strength would decrease more significantly as the replacement rate of SF increases. Additionally, as shown in Figure 8, when the specimen had the same porosity in the range from approximately 13% to 30%, the compressive strength decreased as the replacement rate of SF increased. As shown in Figure 6, as the replacement rate of SF increased, the peaks in the capillary pores and mesopores gradually decreased and increased, respectively. The volume fraction of the large capillary pores has a greater effect on the compressive strength of the cementitious materials than the volume fraction of the small mesopores. Therefore, when the SF-replaced specimen and the reference specimen have the same porosity, the strength of the former should be greater, because the capillary pore-volume fraction of the former is small. However, as shown in Figure 8, the strength tended to decrease as the replacement rate of SF increased. This result is due to the presence of SF agglomerate, which reduces the porosity of the cementitious materials, but from a mechanical point of view, it functions as a large pore. The evidence supporting this fact is the SEM image shown in Figure 9. More specifically, the porosity is reduced by the SF particles acting as a simple filler, which has the effect of dispersing the concentration of the load. However, the SF particles exist in an unhydrated state and are not connected to the hydration products existing around them, as shown in Figure 9, causing them to behave like one large pore when analyzed in terms of mechanical properties. These results were found in the S10 specimen with a low SF replacement rate as well as the S30 specimen with a high SF replacement rate, regardless of age. Therefore, even if the porosity of the specimen is the same, its compressive strength will gradually decrease because the effective porosity with respect to mechanical properties increases with the replacement rate of SF.

### 3.3. Ultrasonic Pulse Velocity (UPV)

**Figure 10:**
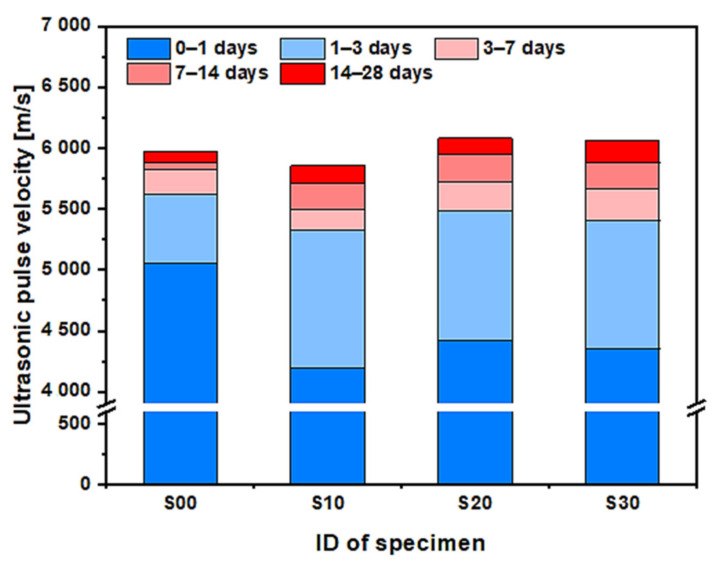
Ultrasonic pulse velocity development of each specimen according to the age range.

Two transducers were placed on both ends of each cylindrical cement mortar specimen, and the UPV (V) was determined by:

(1) V=L/t

where L represents the length of the longitudinal specimen and t is the time taken by the ultrasonic pulse to pass through the specimen. Figure 10 shows the variation in the UPV of each specimen with age. In all specimens, the UPV gradually increases with age, although the greatest rate of increase occurs at an early age, as with compressive strength. The pores filled with moisture are filled with hydration products and, owing to the hydration of cement and the pozzolanic reaction of SF, the volume fraction of the solid phase increases. As a result, areas in the solid phase are connected to each other and the porosity decreases, gradually increasing the UPV. In particular, the UPVs increase in all specimens mostly at an early age as the cement matrix becomes denser, owing to the high heat of hydration and the rapid change in its microstructure. The UPV of the S00, S10, S20, and S30 specimens at 3 days as a percentage of the value at 28 days were 94.2, 90.9, 89.4, and 88.0%, respectively. The chemical reaction between cement and SF slowed such that the UPV increase rate gradually decreased in later ages (refer to Figure 4). As shown in the graph, the UPV of the S00 specimen was the highest until 7 days of age, and the UPV of the S20 specimen was the highest at 14 and 28 days of age. The S10 specimen showed the lowest UPV among all specimens, regardless of age. The difference in the UPV value between different replacement rates of SF was large at an early age; however, the difference tended to decrease gradually with age. As the replacement rate of SF increased, the development of the UPV after the early age showed a tendency to gradually increase. This tendency is closely related to the result of the pozzolanic reaction by SF in which the microstructure becomes denser than that of the S00 specimen (refer to Figure 6).

Since the ultrasonic pulse is transmitted through the solid phases, the UPV of cementitious materials having porous characteristics has a proportional relationship with the volume fraction of the solid phase and an inverse relationship with porosity [26,38]. Therefore, as shown in Figure 5, as the porosity decreases with increased SF replacement rate, the UPV should show a linear relationship as it increases with the SF replacement rate regardless of age. However, the UPV has a nonlinear relationship with the replacement rate of SF, as shown in Figure 10. The UPV of cementitious materials is greatly influenced by the density of the specimen as well as changes in the microstructure owing to the chemical reaction between the cement, SF, and water [21,26]. The density of SF used in this study is approximately two-thirds of that of cement. As shown in Table 2, the amount of water, aggregate, and chemical admixture are all the same except for the binder materials, and the variation in the mixture proportions in this experiment is only in the replacement rate of SF. As all the specimens are cured under a sealed state to prevent moisture transfer to the outside, the density of each specimen has the same value at all ages. Therefore, as the replacement rate of SF increases, the density of the specimen decreases at all ages and, from this point of view, the UPV of the specimen should decrease as the SF replacement rate increases. Additionally, Wang et al. experimentally demonstrated that UPV gradually decreases in pore size in the case of having the same porosity [39]. As shown in Figure 6, the peak in the pore-size distribution of cementitious materials occurred increasingly in the mesopore size range rather than the capillary pore-size range as the replacement rate of SF increased. That is, as the replacement rate of SF increases, the overall pore size of cementitious materials tends to decrease. Thus, because the UPV of cementitious materials is complexly influenced by the porosity, density, and pore size of the specimen, it is judged that the replacement rate of SF and UPV have a nonlinear relationship.

### 3.4. Relationship between Compressive Strength and UPV

**Figure 11:**
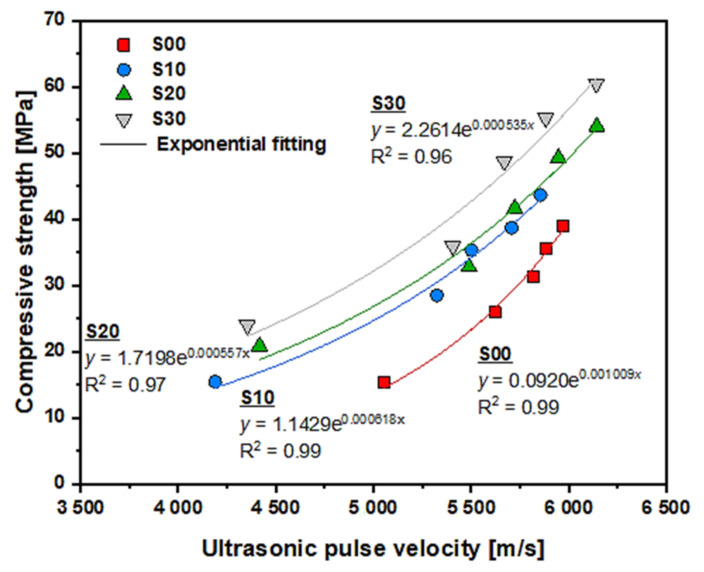
Exponential relationship between UPV and compressive strength according to the replacement rate of SF.

Figure 11 shows the exponential relationship between compressive strength and UPV for 1, 3, 7, 14, and 28 days of age in each specimen. Various studies [22,40,41,42] have shown that the relationship can be estimated by

(2) $$f_{c}=A\cdot e^{B\cdot V}$$

where $f_{c}$ is the compressive strength and A and B are empirical constants. As shown in Figure 11, the compressive strength increased with UPV in all specimens, and the curve shape of each specimen differed. The regression coefficients for A and B and the correlation coefficient $R^{2}$ of each specimen are shown in Table 3. There was a very strong exponential relationship between the compressive strength and UPV, of which the correlation coefficient was higher than 0.9603. Compared with the specimens blended with SF, the S00 specimen showed a relatively steep change in compressive strength, owing to the difference in the development rate of the UPV. As the replacement rate of SF increased, the regression coefficient, A, gradually increased. The regression coefficient, B, rapidly decreased when SF was substituted while continuing to fall gradually with the growing replacement rate of SF. The correlation of the compressive strength and UPV in relation to the replacement of SF and its ratio is complexly affected by various factors, such as the filling effect, the change in microstructure caused by further hydration of the pozzolanic reaction, and density of the SF.

**Figure 12:**
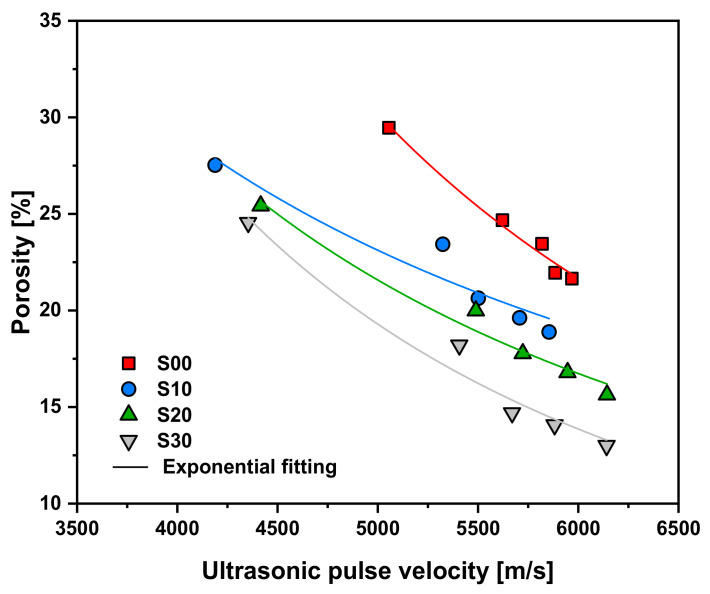
Correlation between UPV and porosity according to the replacement rate of SF.

As seen in Figure 11, when the UPV of the specimen was the same in the range of approximately 4000–6000, the compressive strength increased with the replacement rate of SF. This is closely related to the microstructure of the specimen. Figure 12 shows the correlation between UPV and porosity. As shown in the graph, when the UPV of the specimen was the same within the UPV range of approximately 4000–6000, the porosity decreased as the SF replacement rate increased. Therefore, porosity decreases as the replacement rate of SF increases at the same UPV. Thus, the compressive strength is increased by dispersing the load concentrated in the pores.

## 4. Conclusions

In this study, the correlation between the compressive strength and UPV of cement mortars blended with SF was analyzed for up to 28 days. For this purpose, cement mortars were prepared for varied SF replacement rates, and their compressive strengths and UPVs were measured. Additionally, isothermal calorimetry and MIP experiments were conducted to analyze the hydration kinetics and microstructure, to assess how the replacement rate of SF affects the correlation between compressive strength and UPV. The test results can be summed up as follows:

1. As SF replacement rates increased, the hydration kinetics decreased at early ages, but it increased due to SF’s pozzolanic reaction at a later age. In addition, SF reduced the porosity of the cement mortar by acting as a filler in the pores. The number of mesopores increased and capillary pores decreased, as the content of SF increased.
2. As the SF replacement rate increased, the compressive strength of cement mortar increased. The strength increased because of the filler effect of SF at an early age, and the strength increased through the pozzolanic reaction in combination with the filler effect at later ages.
3. The volume fraction of the solid phase increased as the pores filled with moisture were filled with hydration products with age. The UPV of the specimen gradually increased as the solid phases were connected. The development of UPV showed a nonlinear relationship with the replacement rate of SF, which is closely related to the density and microstructure of the specimen.
4. The UPV and compressive strength of cement mortar have an exponential relationship, which differs by the replacement rate of SF. In general, the substitution of SF had a significant effect on the relationship of the exponential function over the reference specimen; however, variation in the replacement rate of SF was found to have a relatively minor effect.

In previous studies on the relationship between compressive strength and UPV, a correlation analysis was performed for all specimens regardless of the mixture proportion of cementitious materials. The present work evaluated the effect of hydration kinetics and microstructure changes on UPV and compressive strength due to the difference in mixture proportions and examined the correlation between comprehensive strength and UPV for each mixture. The findings from this study can be used to estimate the compressive strength of cementitious materials blended with SF and provide preliminary comparison data for studies that aim to analyze the correlation between the compressive strength and UPV of cementitious materials containing SF and other SCMs.

## Figures and Tables

**Table 1:** Chemical compositions of the binder materials.

Oxide	Cement (mass%)	SF (mass%)
SiO_2_	20.6	93.10
Al_2_O_3_	4.9	0.62
Fe_2_O_3_	3.0	0.41
CaO	61.8	0.66
MgO	2.6	1.16
K_2_O	0.6	–
Na_2_O	0.2	0.67
SO_3_	2.3	<0.01
LOI^1^	2.4	2.71
–	98.8	–

^1^ LOI: loss on ignition.

**Table 2:** Mixture proportions of the specimens (Unit: g).

ID of Specimen	Binder		Sand	Water	Chemical Admixture
	Cement	SF			
S00	1000	–	1000	250	20
S10	900	100			
S20	800	200			
S30	700	300			

**Table 3:** Regression coefficient and least square of each specimen for compressive strength and UPV relationship.

ID of Specimen	Regression Coefficient ( $f_{c}=A\cdot e^{B\cdot V}$)		Correlation Coefficient, $R^{2}$
	A	B	
S00	0.0920	0.001009	0.99
S10	1.1429	0.000618	0.99
S20	1.7198	0.000557	0.97
S30	2.2614	0.000535	0.96

## Data Availability

The data presented in this study are available on request from the corresponding author.
